# A Qualitative Study of Adolescent Views of Sugar-Sweetened Beverage Taxes, Michigan, 2014

**DOI:** 10.5888/pcd13.150543

**Published:** 2016-05-05

**Authors:** Claire N. Krukowski, Kathleen Mullen Conley, Megan Sterling, Alice Jo Rainville

**Affiliations:** Author Affiliations: Kathleen Mullen Conley; Megan Sterling; Alice Jo Rainville, Eastern Michigan University, Ypsilanti, Michigan.

## Abstract

**Introduction:**

We conducted a qualitative study to gather information on adolescent views of how a 20% tax on sugar-sweetened beverages (SSBs) would affect adolescents’ consumption of SSBs. The role of habit in consumption of SSBs was also explored.

**Methods:**

We held 3 focus groups with students from various racial/ethnic groups (N = 22) in grades 6 through 8 at a Michigan middle school. Data on demographic characteristics and beverage consumption were collected. Focus group discussions, guided by the Theory of Planned Behavior, explored adolescent views of a 20% tax on SSBs and the tax’s effect on adolescents’ consumption of these beverages. Focus groups were recorded and recordings transcribed verbatim. Data were coded and analyzed using NVivo software.

**Results:**

Students understood the short- and long-term advantages and disadvantages of drinking SSBs. They understood that the opinions of those around them about SSBs might be affected by personal consumption. Students also understood the personal and economic effects of a 20% tax on SSBs, although the economics of a tax confused some. Students indicated that habit and environment could make reducing consumption of SSBs difficult, but they also gave suggestions, using habit and environment, to reduce consumption. Most students reported that they would decrease their consumption of SSBs if a 20% tax were implemented.

**Conclusion:**

Taxes on SSBs could be used, with other strategies, to reduce adolescents’ high level of SSB consumption.

## Introduction

According to the Centers for Disease Control and Prevention, in 2013, 27% of US high school youth reported that they drank 1 or more soft drinks per day in the past week (1). Another study found that children aged 2 to 16 years who consumed more than 1 sugar-sweetened beverage (SSB) daily were 26% more likely to be overweight or obese (2). SSBs are defined as “any beverage with added sugar or other caloric sweeteners, such as high-fructose corn syrup” (3).

As of January 1, 2014, thirty-four states plus the District of Columbia have implemented SSB taxes in stores, and 5 states tax vending machine sales to raise money and decrease SSB consumption (4). However, it is recommended that the level of SSB taxes be raised to 20% (5–7) or a penny per ounce (8–11), because current levels (<8%) are inadequate to produce significant health benefits. Several studies have explored the impact of current low-level taxes (ranging from 0%–8%) on the body mass index (BMI) of adolescents, using either data from the National Health and Nutrition Examination Survey (12) or data from the Monitoring the Future study (13). These studies confirm that low-level taxes are not sufficient to combat rising levels of adolescent obesity.

We used the Theory of Planned Behavior (TPB) to explore adolescent views of the recommended 20% SSB tax, and possible substitutions adolescents might make when faced with this significantly higher tax. We also investigated the role of habit, because it is a motivator for SSB consumption (14). We used a qualitative study design to gather detailed descriptions of adolescents’ perceptions of the impact of a 20% SSB tax.

## Methods

The College of Health and Human Services’ Human Subjects Review Committee at Eastern Michigan University approved the study protocol. Participants were from a suburban Detroit middle school. The school’s population was 52% female, 54% white, 33% African American, with smaller percentages of other racial/ethnic groups. More than half (56%) of the students were eligible for free or reduced-price lunch. The school was rated as mid-performing on the Michigan School Accountability Scorecard (15), a measure of school achievement. The school’s wellness policy prohibits the sale of SSBs in the school, although SSBs are allowed at parties and dances. SSBs were available for purchase at a gas station near the school.

All teachers with a first-hour class were listed on a Microsoft Excel spreadsheet. The sort feature was used to randomly order teachers’ names. Classrooms were selected in order from the random list until 36 students were recruited. Opt-out letters were mailed to parents in selected classrooms 10 days before the recruitment process began to allow parents to exempt their child from being recruited. Unless a child returned an opt-out letter, assent and consent forms were sent home with students. Active parent/guardian consent was required to assign students to a focus group.

We conducted 3 focus groups in June of 2014. Of 36 students recruited, 22 (61%) attended a focus group. After giving assent, students answered questions about demographic characteristics (Appendix A) and SSB consumption. We used a screener developed by Nelson and Lytle (16) and adapted for this study (Appendix B) to measure the frequency of consumption and amount. The original screener was tested for reliability through test and retest of 33 students, yielding Spearman correlations with κ statistics > .60. Criterion validity was established by comparing results from the SSB screener to 24-hour dietary recall data. Correlations showed a fair level of agreement and were significant (*P* < .002). We adapted the screener by changing the word “soda” to “pop,” a more common term in Michigan. The focus group script followed TPB constructs, exploring attitudes, subjective norms, behavioral intentions, and perceived behavioral control about SSBs and a 20% SSB tax (Appendix C). Questions about SSB habits were also asked. The first author moderated the focus groups with assistance from a note taker. Focus groups were audio-recorded. Students received snacks and a jeans and hoodie pass that temporarily exempted them from the dress code for participating.

We used SPSS version 21 (IBM Corporation) to examine frequency data for demographics, beverage consumption, and quantity. The first author (C.N.K.) transcribed focus group recordings verbatim. The first and second authors independently read the transcripts several times to identify potential codes. The authors discussed the codes to identify overarching themes related to TPB constructs and habit. Additional codes were identified as data queries, including cost, home environment, and taste. All quotes were then encoded and analyzed using QSR NVivo software version 10 (QSR International). Subthemes were identified and interpreted (Figure). The 2 authors discussed any disagreements about themes or subthemes until consensus was reached.

**Figure:**
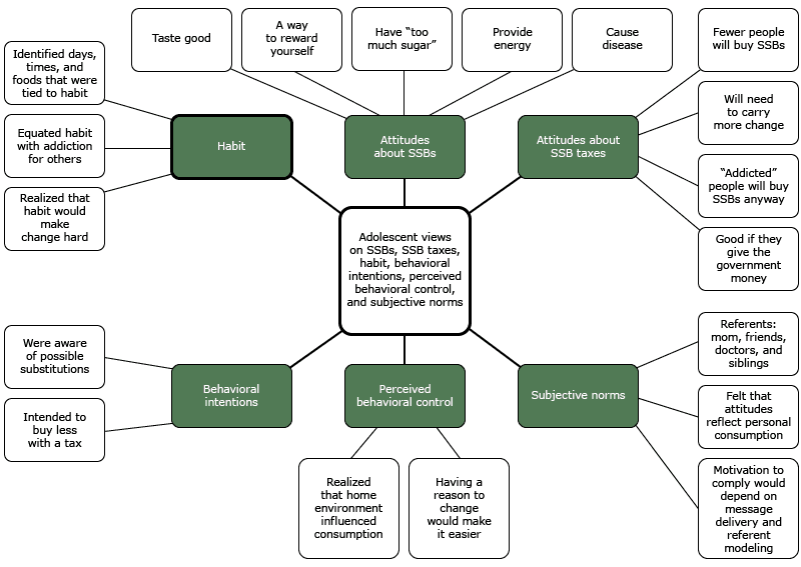
Adolescents’ perceptions of the impact of a 20% sugar-sweetened beverage (SSB) tax, Romulus, Michigan, June 2014

## Results

Of the 22 focus group participants, 13 (59%) were girls and 11 (50%) were white (Table 1). Students reported the frequency of consumption of SSBs and non-SSBs (Table 2), as well as their consumption level for each beverage (Table 3).

**Table 1:** Number and Percentage of Students (N = 22), by Sex and Race/Ethnicity, Focus Group on Consumption of Sugar-Sweetened Beverages, Romulus, Michigan, June 2014

Demographic	No. (%)
**Sex**	
Male	9 (40.9)
Female	13 (59.1)
**Race/ethnicity**	
White	11 (50.0)
Latino	4 (18.2)
African American	3 (13.6)
Other^a^	4 (18.2)

a For “other,” 2 students wrote in “Caucasian/African American” and one wrote “Albanian. The fourth student chose “other,” but did not write in a race/ethnicity.

**Table 2:** Frequency of Students’ (N = 22) Consumption of Sugar-Sweetened Beverages (SSBs) and Non-SSBs, Romulus, Michigan, June 2014

Frequency	SSBs					Non-SSBs^b^		
	Pop^a^, n (%)	Coffee, n (%)	Energy Drink, n (%)	Sports Drink, n (%)	Other^b^, n (%)	Diet Pop^a^, n (%)	Milk, n (%)	Water, n (%)
Rarely or never	3 (13.6)	14 (63.6)	13 (59.1)	4 (18.2)	3 (13.6)	18 (81.8)	4 (18.2)	0
1 time per month	3 (13.6)	0	5 (22.7)	5 (22.7)	2 (9.1)	0	0	0
2 to 3 times per month	4 (18.2)	3 (13.6)	1 (4.5)	3 (13.6)	4 (18.2)	1 (4.5)	2 (13.6)	0
1 to 2 times per week	5 (22.7)	2 (9.1)	2 (9.1)	4 (18.2)	4 (18.2)	1 (4.5)	3 (13.6)	1 (4.5)
3 to 4 times per week	5 (22.7)	2 (9.1)	1 (4.5)	4 (18.2)	4 (18.2)	0	1 (4.5)	1 (4.5)
5 to 6 times per week	1 (4.5)	1 (4.5)	0	2 (9.1)	3 (13.6)	1 (4.5)	4 (18.2)	4 (18.2)
1 time per day	1 (4.5)	0	0	0	1 (4.5)	0	4 (18.2)	0
2 times per day	0	0	0	0	0	1 (4.5)	1 (4.5)	1 (4.5)
3 times per day	0	0	0	0	1 (4.5)	0	3 (13.6)	15 (68.3)

Abbreviations: SSB, sugar-sweetened beverages.

a “Pop” is the regional word for “soda” in Michigan.

b “Other” beverages were described as “sweetened beverages like sweetened tea, juice boxes, punch, or lemonade.”

**Table 3:** Amount of Sugar-Sweetened Beverages (SSBs) and Non-SSBs Consumed by 22 Student Focus Group Participants, Romulus, Michigan, June 2014

Measure	Sugar-Sweetened Beverages					Non-SSBs		
	Pop^a^, n (%)	Coffee, n (%)	Energy Drinks^b^, n (%)	Sports Drinks, n (%)	Other^c^, n (%)	Diet Pop^a^, n (%)	Milk, n (%)	Water, n (%)
I don’t drink it	2 (9.1)	11 (50)	15 (68.2)	3 (13.6)	5 (22.7)	15 (68.2)	4 (18.2)	2 (9.1)
One container	18 (81.9)	8 (36.4)	6 (27.3)	14 (63.6)	9 (40.9)	5 (22.7)	9 (40.9)	1 (4.5)
More than one container	2 (9.1)	3 (13.6)	0	5 (22.7)	8 (36.4)	2 (9.1)	9 (40.9)	19 (86.4)

a “Pop” is the regional word for “soda” in Michigan.

b Values do not add up to 100% (1 student did not answer this question).

c “Other” beverages were described as “sweetened beverages like sweetened tea, juice boxes, punch, or lemonade.”

- Twelve students (54%) reported consuming pop at least once per week; 6 (32%) reported drinking pop on 3 or more days per week. Thirteen students (59%) reported consuming “other” SSBs, including sweetened tea, lemonade, and juice that is not 100% fruit juice, at least once per week; 9 (41%) reported drinking “other” SSBs on 3 or more days per week. Ten students (46%) reported consuming sports drinks at least once per week; 6 (27%) reported drinking sports drinks on 3 or more days per week.
- Water consumption was higher than any other beverage. All students reported drinking water at least once weekly, and 15 students (68%) reported drinking it 3 or more times daily. Nineteen students (86%) reported drinking more than 1 container each time they consumed water.
- Diet pop, coffee, and energy drinks were “rarely or never” consumed by 18 students (82%), 14 students (64%), and 13 students (59%), respectively.

Focus group results were summarized according to themes guided by the TPB and habit (Figure). Many students shared positive attributes of SSBs, including providing energy (“They have sugar in them but they keep you awake.”) and tasting good (“I think that they’re delicious.”). A few mentioned wanting to support companies that make SSBs and stores that sell them (“People buy them and that's how people make money. So I support them.”). The negative attributes mentioned included having “too much sugar” and making the consumer “hyper” and “fat.”

Many students stated that a positive of SSB taxes was decreased consumption (“I think it would stop so many people from buying sugary drinks.”). Two focus groups discussed who would receive the tax money: the government, stores selling SSBs, or the pop industry. A student said, “Pop companies would make more money, so I support them. They would make more money, and they would produce more pop.” Another student corrected him: “The tax goes to the government, which would give them [sic] money to fix our broken streets.”

Many students mentioned that they discuss health issues with their mothers and doctors. Grandparents and siblings were also frequently mentioned; fathers were mentioned much less frequently. Friends were rarely mentioned in the context of health. However, opinions of both friends and parents were important regarding purchasing SSBs. One group said that friends were more important because “kids listen to their friends more than their parents.” Another stated, “Well, anyone that really you look up to or that inspires you [is influential], because if they are telling you something . . . you are probably going to say, OK, I am going to take this from you.” When asked whose opinions they would not listen to about SSB consumption, many students mentioned siblings and friends because “they might be addicted to it and probably would like to get you used to drinking it.” Parents were also mentioned as people they would not listen to because “they yell and make you feel bad.”

The students believed that all referents thought that drinking fewer SSBs was positive, unless the referents drink SSBs frequently. Students stated, “Your family might not care because they’re drinking them” or “my friends would say that it’s OK because that’s what everyone does now.” When asked about what the referents’ feelings would be about an SSB tax they responded, “I don’t think that people are going to really think about it because they are addicted.” Regarding people such as doctors, whom students believed would be against adolescents’ drinking SSBs, they stated, “They would think that it [a SSB tax] is a good idea.”

Students seemed to understand how changing the environment could decrease SSB consumption. An individual could consume fewer SSBs by “getting rid of what’s in your fridge.” Students realized that their home environment and the placement of foods in their homes affected consumption. A student said, “Usually the healthy [drinks] are in the back and the not-so-healthy drinks are in the front. So whatever is in the front is what I usually grab.” Another mentioned, “Today, if I’ve been drinking water all day and like tomorrow my dad brings home dozens of pops, I’ll literally devour the pop. And I’ll forget all about the water.”

When asked what they would do if there was a 20% SSB tax in real life, many students said that they would drink fewer SSBs, with several noting that it would be “too expensive.” When asked why they might continue to drink SSBs with a 20% tax, some students said personal enjoyment or because of its cost relative to other drinks. One student stated, “I will probably still do the reusable water bottle, but I would probably still buy it [an SSB] a lot because they are still cheaper than anything healthy, which is kind of dumb.” Others mentioned using SSBs as a reward “like, after a hard day at school.”

When asked how high a tax would have to be for them to stop drinking SSBs, students answered 50% or 100%. When discussing the impacts of price increases, some students believed that pop companies would close. Others believed companies would just increase prices. The students in one group stated, “Nobody’s going to pay for something that’s $4 for a can of pop or a tiny bottle of pop.” Another responded, “Then soda companies would start going out of business as well.” Another student disagreed, “No, they’ll jack up the price.” Another student concluded, “I think that the only people that will actually do that [continue to buy SSBs] are people that are really rich and can afford anything or people that just don’t know how to handle an addiction of theirs.”

Students noted several ways that they would drink water as a substitute for SSBs: as water with flavored inserts or water with fruit in it. Several students indicated that they would ease into it, suggesting they would “just buy the powdered lemonade and just add a little bit less lemonade, the powder, every time.”

Students seemed to understand the concept of habit, defining it as “If you do it a lot, it would be habit.” Many students stated that, for some people, the 20% SSB tax would not make a difference because some people are “addicted.” One student noted, “They are probably going to buy them whether or not they cost a lot of money.” Several others equated SSBs to a cigarette habit, stating, “Pop is addictive just like grown-ups get addicted to cigarettes.” Students mentioned holiday events and eating at restaurants as triggers for SSB consumption. Several students mentioned sports practices or being outside in warm weather as times when they consume SSBs “to keep the electrolytes in our bodies.”

Students seemed to understand how habit would make decreasing SSB consumption both easy and hard. One student suggested that one could decrease consumption by “find[ing] new healthier habits such as working out, or drinking water, or taking a run or a walk with your dog or pet.” Several others mentioned habit likewise could make it difficult to give up SSBs since they are in the habit of buying it, explaining, “Umm, for some stubborn people and some obese people, they probably wouldn’t want to get away from it. And it would probably take a while for them to get used to something new.”

## Discussion

This is the first known study exploring adolescent views on a recommended 20% SSB tax. Results have implications for intervention strategies targeting the reduction of adolescent SSB consumption through a recommended 20% SSB tax and other health promotion strategies.

Students mentioned taste as an advantage, as found in other studies (17–19). Students recognized the same top 3 health risks of SSBs that adults (20) and other students (17,18) mentioned in prior research. Consumption levels, despite the health risks, indicate that although students understood the disadvantages of SSBs they, like adults, continued to drink them.

Students saw many of the same advantages and disadvantages of a 20% SSB tax that adults did (21). They recognized that a tax on SSBs could decrease consumption and fund the government with money to provide services. They recognized some of the same disadvantages of price increases to individuals and businesses (21). Some students perceived disadvantages that were more short-term. For example, they did not like the idea that they would have to carry more change to drink an SSB.

Although mothers and friends were most frequently mentioned as referents, other family members and doctors were also included. Most other studies of soda consumption have also used parents (22,23) and friends (24) as the assumed referents.

Parents and friends were both mentioned as referents whose SSB advice might be followed or rejected, depending on the context. Students appeared willing to follow a referent’s advice to drink fewer SSBs, but only if that advice was modeled by the referent and if it was delivered in the correct way. Parental modeling has been found to impact SSB consumption in adolescents in other studies (19,22,23); one study found that children aged 8 to 13 were almost 3 times as likely to consume SSBs regularly if their parents did (19). Students shared personal stories as examples of advice they would follow, such as a parent sharing how pop consumption contributed to her diabetes or the choir teacher saying that dehydration from drinking pop would cause them to not sing as well. Further research is needed on the effects of referent communication style with adolescents as a way to decrease SSB consumption.

Many students said the proposed 20% SSB tax would make consuming SSBs “too expensive.” Some students indicated that taxes would have to be higher, as high as 100%, to cause them to eliminate SSBs. Similar results were found with cigarette taxes (25). Most adolescents are sensitive to price and smoke less when cigarette price increases. A smaller group of adolescents are not responsive to increased price, especially those with low self-control. Further research on adolescents’ perceived self-control as related to the effect of a significant (20%) SSB tax would provide valuable insights for policy and other health promotion interventions.

Although students recognized that they sometimes bought their own SSBs, they most often referred to SSBs bought by their parents. They felt that without adult support, personal changes would be more difficult. This supports findings from prior studies that the largest predictor of perceived behavioral control, a person’s beliefs that they have control over a behavior, was the availability of SSBs in the home (17,18). Perceived behavioral control or self-efficacy is one of the top predictors of SSB consumption (22,26). Parenting practices, such as limiting access by telling adolescents which SSBs and how much of the SSB to drink, have been effective in lowering the adolescents’ consumption (19,22,24). More than two-thirds of students report that they would drink another beverage if there were no soft drinks in the home (17,18). Students in this study indicated that a decreased availability of SSBs in the home or what was kept cold would decrease their consumption. This suggests that an intervention targeting parents, instructing them about the placement and availability of SSBs in the home, may be beneficial in decreasing adolescent consumption of SSBs.

Habit is stronger than (23,26) or as strong as (22) some constructs of the TPB in predicting SSB consumption. Students seemed to understand the role that habit plays in SSB consumption. They mentioned that changing routines, such as doing something else or drinking something else, would affect their consumption of SSBs. Although students recognized that they had a habit of drinking SSBs, they usually used the word “addicted” to refer to other people, not themselves. This may be accurate, as these students seemed to consume fewer SSBs than average. In this study, one student reported drinking soda or pop daily compared with 19.6% and 27% of Michigan and US ninth-graders, respectively (27). This study’s low consumption level and references to pop bought by parents may be due to students being unable to purchase SSBs in school (28).

These findings should be interpreted with caution, because participants were drawn from one school and results may be unique to that school. Additionally, because of limitations inherent in the SSB screener, we could not compute an overall rate of SSB consumption. Each response category gave a range of times or amounts, rather than a single time or amount that could be added for an overall consumption rate (Appendix B). Students in this study consumed less pop than nationally reported averages; however, it is possible that students’ total SSB consumption was higher. Additional studies are needed to assess overall SSB consumption of adolescents.

This study is unique in that it qualitatively studied adolescent views on SSBs in the context of a significant (20%) SSB tax. Many students in this study reported that they intended to buy less SSBs if a 20% tax was implemented. Additionally, our results suggest that changes in the home environment and parental communication strategies might be effective ways to decrease adolescent SSB consumption.

## Appendices

Appendix A. Demographic Survey

Appendix B. Sugar-Sweetened Beverage Consumption Screener

Appendix C. Focus Group Questions, Romulus, Michigan, June 2014

These appendices are available for download as a Microsoft Word Document.
