# Supplementary material for: A Qualitative Study of Adolescent Views of Sugar-Sweetened Beverage Taxes, Michigan, 2014
Source: Prev Chronic Dis. 2016 May 5;13:E60. doi: 10.5888/pcd13.150543 (PMC4858450; doi:10.5888/pcd13.150543)
Supplement: Supplementary file 1 [file 15_0543_Appendices.docx]

**Appendices**

**Appendix A: Demographic Survey**

**A Little About You**

Directions: Circle the answer that best describes you.

Gender: Male Female

Race: African-American/Black White/Caucasian Latino/Hispanic Asian

I would rather not say. Other: ________________

**Appendix B: SSB Screener**

Directions: Circle the answer that best matches the number of times that you drink the beverages below.

| **How often do you drink regular pop (including Slurpees), not including diet pop?**  Never or rarely 1 time per month 2 to 3 times per month  1 to 2 times per week 3 to 4 times per week 5 to 6 times per week  1 time per day 2 times per day 3 or more times per day |
| --- |
| **How often do you drink diet pop?**  Never or rarely 1 to 2 times per week 1 time per day  1 time per month 3 to 4 times per week 2 times per day  2 to 3 times per month 5 to 6 times per week 3 or more times per day |
| **How often do you drink sweetened coffee drinks (not including sugar-free sweeteners or syrups)?**  Never or rarely 1 to 2 times per week 1 time per day  1 time per month 3 to 4 times per week 2 times per day  2 to 3 times per month 5 to 6 times per week 3 or more times per day |
| **How often do you drink energy drinks like Monster?**  Never or rarely 1 to 2 times per week 1 time per day  1 time per month 3 to 4 times per week 2 times per day  2 to 3 times per month 5 to 6 times per week 3 or more times per day |
| **How often do you drink sports drinks?**  Never or rarely 1 to 2 times per week 1 time per day  1 time per month 3 to 4 times per week 2 times per day  2 to 3 times per month 5 to 6 times per week 3 or more times per day |
| **How often do you drink milk (not in your cereal)?**  Never or rarely 1 to 2 times per week 1 time per day  1 time per month 3 to 4 times per week 2 times per day  2 to 3 times per month 5 to 6 times per week 3 or more times per day |
| **How often do you drink other sweetened beverages like sweetened tea, juice boxes, punch or lemonade?**  Never or rarely 1 to 2 times per week 1 time per day  1 time per month 3 to 4 times per week 2 times per day  2 to 3 times per month 5 to 6 times per week 3 or more times per day |
| **How often do you drink water?**  Never or rarely 1 to 2 times per week 1 time per day  1 time per month 3 to 4 times per week 2 times per day  2 to 3 times per month 5 to 6 times per week 3 or more times per day |

Directions: Think about the last time that you drank the beverages listed below. Then pick the answer the best matches the amount that you drank.

| **The last time that you drank pop, including Slurpees™, but not diet pop, how much did you drink?**  I don't drink this. One container or less More than one container |
| --- |
| **The last time that you drank diet pop, how much did you drink?**  I don't drink this. One container or less More than one container |
| **The last time that you drank sugar sweetened coffee drinks, how much did you drink?**  I don't drink this. One container or less More than one container |
| **The last time that you drank energy drinks like Monster™, how much did you drink?**  I don't drink this. One container or less More than one container |
| **The last time that you drank sports drinks, how much did you drink?**  I don't drink this. One container or less More than one container |
| **The last time that you drank milk (not on your cereal), how much did you drink?**  I don't drink this. One container or less More than one container |
| **The last time that you drank other sweetened beverages like sweetened tea, juice boxes, punch or lemonade, how much did you drink?**  I don't drink this. One container or less More than one container |
| **The last time that you drank water, how much did you drink?**  I don't drink this. One container or less More than one container |

**Appendix C. Focus Group Questions for the 3 Focus Groups, Romulus, Michigan, June 2014**

| Attitude | - What do you think about SSBs? - What do you think about a new tax on sugar-sweetened beverages like I just showed you? |
| --- | --- |
| Subjective norms | - Who are some people that you talk to about health related things? - What do you think that the people written on the white board think about adolescents drinking SSBs? - How do you think these people's opinions might change about teens drinking SSBs if the tax went up? - Now I would like you to think about which of the people written on the board might influence your SSB buying. Whose opinions about SSB buying, if anyone, would most likely change the amount of SSBs that you buy? - Whose opinions, if anyone, would make you do the opposite of what they recommend? |
| Intentions | - If the price of sugar-sweetened beverages went up 20% like is shown on the bottles over there, what would you do? |
| Perceived behavioral control | - If at some point, SSBs became too expensive to buy, what would you do? - What other types of drinks or food might you buy instead, if any? - What would help make a change like that easy? - What would make it hard? |
| Habit | - Are there times that habit influences you to drink sugar-sweetened beverages? - Are there ways that habit might make it difficult to drink less or no sugar sweetened beverage? - Are there ways that habit might make it easy to drink less or no sugar sweetened beverages? |
